# Comparison of the Parasitization of *Chelonus inanitus* L. (Hymenoptera: Braconidae) in Two *Spodoptera* Pests and Evaluation of the Procedure for Its Production

**DOI:** 10.3390/insects13010099

**Published:** 2022-01-15

**Authors:** Antonio Jesús Magaña, Beatriz Dáder, Gonzalo Sancho, Ángeles Adán, Ignacio Morales, Elisa Viñuela

**Affiliations:** Unidad de Protección de Cultivos, Escuela Técnica Superior de Ingeniería Agronómica, Alimentaria y de Biosistemas, Universidad Politécnica de Madrid (UPM), Avda. Puerta de Hierro 2, 28040 Madrid, Spain; aj.magana@alumnos.upm.es (A.J.M.); g.sancho@creaf.uab.cat (G.S.); angeles.adan@upm.es (Á.A.); i.morales@upm.es (I.M.); elisa.vinuela@upm.es (E.V.)

**Keywords:** biological control, *Ephestia kuehniella*, lepidoptera, *Spodoptera exigua*, *Spodoptera littoralis*

## Abstract

**Simple Summary:**

Successful integrated pest management of horticultural crops requires the evaluation of natural enemies used in biological control. To improve the mass rearing of *Chelonus inanitus* (L.) in the factitious host *Ephestia kuehniella* Zeller, we investigated the influence of host age and number of females parasitizing simultaneously on quality parameters evaluated in the commercial production of biological control agents, such as overall rearing success, duration of life cycle and body size. Under semi-field conditions, we investigated how the competition among females impacted the parasitism success on important pests *Spodoptera exigua* (Hübner) and *S. littoralis* (Boisduval). For the parasitoid mass rearing optimization, mature eggs seem to be more convenient because they increased the female percentage in the offspring and shortened the parasitoid life cycle. Furthermore, a high number of females parasitizing simultaneously reduced the emergence of non-parasitized hosts. Under more realistic conditions, the parasitoid effectively controlled both pests, but *S. exigua* may be a more convenient host because the parasitoid offspring was much larger. With this research, we provide a foundation that aims to better manage these cosmopolitan, highly polyphagous pests that exhibit long-distance migratory potential, alongside the potential use of this natural enemy.

**Abstract:**

*Chelonus inanitus* (L.) is an egg-larval parasitoid of noctuids *Spodoptera exigua* (Hübner) and *S. littoralis* (Boisduval), whose mass rearing or real potential has not been targeted yet. To improve the rearing in the factitious host *Ephestia kuehniella* Zeller, we investigated the influence of host age and number of females parasitizing simultaneously on the overall rearing success, the influence of host age on the life cycle, and the influence of host species on the parasitoid body size. The proportion of emerging *C. inanitus* was higher from young host eggs, but more females emerged from mature eggs. Under high parasitoid competition, we observed a reduction in non-parasitized hosts without reducing parasitoid emergence. The parasitoid life cycle was longer in females, but the mismatch between sexes was smaller in mature eggs. The parasitoid size was smaller in the factitious host than in the natural hosts. Under semi-field conditions, we investigated the competition among parasitoid females on the overall parasitism success. The reproductive parasitism was more successful in *S. exigua* than in *S. littoralis*, and the maximum emergence was reached with three and four females, respectively. The control of *S. littoralis* may be attributed to the high developmental mortality, a non-reproductive parasitism that is often underestimated.

## 1. Introduction

*Spodoptera* pests (Lepidoptera: Noctuidae) are highly feared in many crops globally. They are highly polyphagous, can be found on almost all types of commodities of plants and exhibit a long-distance migratory potential [1]. The cosmopolitan species, *S. exigua* (Hübner) (the beet armyworm) is present in most European and Mediterranean Plant Protection Organization (EPPO) countries, including Spain, attacking vegetables, cotton and ornamental plants, both outdoors and protected. In the greenhouses of Southeastern Spain, it is a key pest in tomato (*Solanum lycopersicum* L.) and sweet pepper (*Capsicum annuum* L.) [2,3,4,5]. *Spodoptera littoralis* (Boisduval) (the cotton leafworm), included in the EPPO A2 quarantine list [6], lives in the Palearctic region and can attack over 40 families of plants containing about 87 species of economic importance [1,7,8,9]. This species is also present in the protected crops of Southeastern Spain [4].

Because Integrated Pest Management (IPM) programs have been mandatory in the EU since 2014 [10], several ecologically acceptable tools for controlling *Spodoptera* are available in Spain (e.g., some nematodes and pheromone mating disruption). However, due to their lack of efficacy, farmers use pesticides with different modes of action, such as cyantraniliprole, indoxacarb, methoxyfenozide, tebufenozide, metaflumizone, deltamethrin, SpliNPV (*S. littoralis* nucleopolyhedrovirus) and *Bacillus thuringiensis* Berliner [11,12]. However, the control provided by these pesticides is seriously compromised due to the rapid global development of resistance towards a wide range of pesticides, including not only organophosphates and pyrethroids, but also active ingredients with low toxicity, high activity, novel modes of action and low environmental safety (e.g., chlorantraniliprole, emamectin benzoate, spinosad, tebufenozide) [13,14,15,16,17].

Both *S. exigua* and *S. littoralis* have a long list of natural enemies, including arthropods and entomopathogenic viruses, bacteria and fungi [7,8,14]. In Spain, the few commercially available arthropods [18] are not efficient enough to counterbalance the population growth of these pests, especially inside greenhouses, where the use of pesticides is still high [19,20]. Therefore, there is an urgent need to identify effective biological control agents for these noctuids.

The subfamily Cheloninae (Family Braconidae) harbors a group of over 700 species of egg-larval solitary koinobiont endoparasitoids of Lepidoptera. They oviposit inside the host eggs, which follow development until the beginning of the third larval instar, the moment at which the parasitoid induces a precocious onset of the metamorphosis and exits to spin a silken cocoon and pupate [21,22]. The species *Chelonus inanitus* (L.) and *C. oculator* (F.) have been cited in Spain as frequent parasitoids of *S. exigua* and *S. littoralis* [14,23], but they are not commercially available at present [18].

The cosmopolitan species *C. inanitus* is present in four continents: America (USA, Canada), Asia (Israel), Africa (Egypt) and Europe [24], where it attacks about 22 species of Lepidoptera in the families Noctuidae, Pyralidae, Tortricidae and Gelechiidae [25]. Under laboratory conditions, it can complete its development in several factitious hosts and can easily be mass reared with lower economic cost: *Ephestia* (*Anagasta*) *kuehniella* Zeller (Lepidoptera: Pyralidae), *Cadra cautella* (Walker) (Lepidoptera: Phycitinae) and *Sitotroga cerealella* (Olivier) (Lepidoptera: Gelechiidae) [21]. Nevertheless, this parasitoid undergoes arrhenotoky reproduction [26] and the male percentage can become rapidly biased in a few generations, which is a key problem for mass rearing [27].

A large portion of laboratory studies has focused on different aspects of *C. inanitus* physiology: polydnavirus and venom production [28,29], effects on host nutritional physiology [30,31] and endocrine interactions [32,33]. However, the optimization of its mass rearing or the evaluation of its real potential as a biological control agent of *S. exigua* and *S. littoralis* has not been targeted yet.

The aim of our study was to gain knowledge on *C. inanitus* regarding two facets: (1) to improve the rearing in the factitious host *E. kuehniella* by studying the influence of different host ages and the number of females parasitizing simultaneously on the parasitoid mass rearing success; and (2) to investigate the parasitoid efficacy for the control of *S. exigua* and *S. littoralis* under semi-field conditions.

## 2. Materials and Methods

### 2.1. Insect Rearing

A laboratory colony of *C. inanitus* was initiated with specimens kindly provided by the Institute of Cell Biology of the University of Bern (Bern, Switzerland) and continuously mass reared on *E. kuehniella* following a procedure developed in our facilities. The entire rearing process was completed at a 20–24 °C temperature, 60% RH and 16L:8D photoperiod, inside a climate chamber (Sanyo MLR-350, Sanyo Electric Co. Ltd., Osaka, Japan). *Ephestia kuehniella* eggs, collected by introducing adults into a transparent plastic box (10 × 17 × 7 cm) for 24 h, were pasted to filter paper with tragacanth gum (Manuel Riesgo S.A., Madrid, Spain), and introduced into the ventilated general rearing cage (40 × 30 × 30 cm) of *C. inanitus* adults for 90 min to allow parasitism. Then, the filter paper was removed and placed in a ventilated transparent methacrylate cage (16 × 25 × 5 cm) containing 290 g wheat flour (Comercial Gallo S.A., Córdoba, Spain) and 10 g brewer’s yeast (Biogran S.L., Madrid, Spain). The box was maintained inside the climate chamber until the emergence of adults. Adults were fed with a 70% honey (Aljara flor, Andaluza de Mieles S.L., Sevilla, Spain) solution in distilled water.

The colonies of *S. exigua* and *S. littoralis* were started with larvae provided by the Universidad Pública de Navarra (Pamplona, Spain) that originally came from populations collected in the protected vegetable crops of Almería (Spain). Larvae were continuously reared on a semi-solid wheat germ-based semi-synthetic diet, slightly modified [34], in ventilated transparent plastic boxes (30 × 20 × 10 cm), inside a walk-in chamber at 25 ± 2 °C, 45 ± 1% RH and 16L:8D photoperiod. After pupation, adults were introduced into ventilated methacrylate cages (40 × 30 × 30 cm) and fed with a 50% honey solution. Filter paper was provided as an oviposition substrate and periodically replaced, as required.

### 2.2. C. inanitus Development in the Factitious Host E. kuehniella

To optimize *C. inanitus* mass rearing under laboratory conditions, we firstly studied the influence of the host age and the number of parasitoid females on *C. inanitus* development, by evaluating the percentages of *C. inanitus* adults per offered eggs (reproductive parasitism), *C. inanitus* females per emerged parasitoids (sex ratio), and *E. kuehniella* adults per offered eggs in the offspring. In addition, the developmental mortality was evaluated as the difference between 100 and the sum of *C. inanitus* and *E. kuehniella* adults emerged. Groups of 200 *S. exigua* eggs were offered to *C. inanitus* females for 90 min because females spend about 5.5 s per *S. littoralis* egg for parasitization [26]. In our laboratory rearing, we observed that interruptions in the parasitization process were more frequent when 5 or more females were attacking the same egg patch. Therefore, we studied two different numbers of females parasitizing simultaneously, 1–3 females, low competition during parasitization; and 5 females, high competition; and two different host ages: young, 1-day-old eggs; mature, 4-day-old eggs. The four treatments considered were: young eggs parasitized either by a low or a high number of females and mature eggs parasitized either by a low or a high number of females (*n* = 10 replicates per treatment).

In a second trial, the duration of the parasitoid cycle depending on the host age was studied, by offering 50 young (1-day-old) or mature (4-day-old) *E. kuehniella* eggs for 90 min to individualized *C. inanitus* females. A lower number of eggs compared to the previous experiment was offered in order to be able to follow the emergence of every parasitoid adult. The cycle duration of non-parasitized eggs of both ages was also followed (*n* = 10 replicates for young eggs and *n* = 9 replicates for mature eggs).

In a third trial, we measured the parasitoid body size when developed in natural and factitious hosts. Even though in koinobiont species the parasitoid progeny is not correlated to the original amount of food, we wanted to ascertain whether the parasitoid development in the factitious host *E. kuehniella* can cause any alteration to the size of the parasitoid, which could compromise its biological performance in comparison with the development in two natural hosts, *S. littoralis* and *S. exigua*. Two hundred eggs (2-day-old) of *S. exigua*, *S. littoralis* or *E. kuehniella* were offered to 3 *C. inanitus* females for 90 min for parasitization (*n* = 4 replicates per host species). Adults were immediately killed after emergence with ethyl acetate, and the body length from the point of the head to the tip of the abdomen along the longitudinal axis of 10 males and 10 females per replicate were measured under binoculars.

### 2.3. Semi-Field Studies on the Efficacy of C. inanitus in S. exigua and S. littoralis 

To evaluate the parasitoid performance under more realistic conditions, 5-true-leave sweet pepper potted plants (*Capsicum annuum* L. cv. “Dulce Italiano”) were placed inside cylindrical ventilated plastic cages (12.5 cm diameter by 20 cm height). Ten unsexed lepidopteran adults were allowed to mate and oviposit for 48 h. Cotton soaked in 50% honey solution was provided in small plastic vials. The next day, adults were removed and 60 eggs from randomly selected patches were left per plant, after cleaning them with a brush because eggs were covered with hairs from the female abdomen [1]. Then, a different number (1, 2, 3 or 4) of experienced parasitoid females (>72 h old) were introduced into the cages and allowed to parasitize the eggs for 24 h. The leaves with eggs were cut, the petiole introduced into an Eppendorf tube with agar to maintain leaf turgidity and placed inside round ventilated plastic cages (12 cm diameter by 5 cm height) until egg hatching under laboratory conditions (see Section 2.1). Larvae were then moved to bigger ventilated cages (19 × 16 × 9 cm) and provided with the same artificial diet used for rearing. The impact of the number of parasitoid females on the number of *C. inanitus* adults emerged per offered eggs (reproductive parasitism), the number of *Spodoptera* adults emerged per offered eggs, and the developmental mortality were recorded. Controls with non-parasitized eggs were included in order to compare the normal host development (*n* = 6 replicates per treatment). Experiments were performed in autumn (23.3 ± 0.22 °C, 59.9 ± 0.63% RH, natural photoperiod).

### 2.4. Statistical Analysis

All data are presented as mean ± SEM, and were analyzed by analysis of variance (ANOVA, *p* < 0.05) using Statgraphics Centurion 18 version 18.1.06 (The Plains, VA, USA) [35]. Experiments performed in the factitious host under laboratory conditions were analyzed with a two-way ANOVA in order to determine the main effects of the factors and their interaction. In (1) *C. inanitus* development, the two factors were the number of females parasitizing simultaneously and host age; in (2) the factors were duration of *C. inanitus* cycle, host age and parasitoid sex; and in (3) the factors were *C. inanitus* body size, host species and parasitoid sex. When the interaction was significant, pairwise comparisons were run thereafter to identify the simple main effects at a confidence interval adjustment of the least significant difference (LSD). Data on developmental mortality were transformed to arcsin √(x/100) prior analysis to meet the requirements of homoscedasticity. Semi-field assays with the two natural hosts were performed sequentially. Therefore, in every *Spodoptera* species, the percentage of *C. inanitus* adults, the host percentage that escaped parasitism, and the developmental mortality were analyzed with a unifactorial ANOVA.

## 3. Results

### 3.1. C. inanitus Development in the Factitious Host E. kuehniella

The percentage of *C. inanitus* adults emerged (reproductive parasitism) oscillated between 34 and 57% (Table 1). There was a statistically significant interaction between the number of females parasitizing simultaneously (one–three, low competition; five, high competition) and the host age (young, 1-day-old eggs; mature, 4-day-old eggs) (*F*_1,36_ = 6.80, *p* = 0.01). However, the interaction effect was not conclusive and the pairwise comparisons only detected a significant effect on the host age when five females parasitized simultaneously. In contrast, the percentage of *C. inanitus* females emerged was dependent on the host age (*F*_1,36_ = 5.67, *p* = 0.02) and it was higher when females parasitized mature eggs (Table 1). The percentage of *E. kuehniella* adults in the offspring was significantly different depending on the number of *C. inanitus* females parasitizing simultaneously (*F*_1,36_ = 17.80, *p* < 0.001) (Table 1), and there was not any interaction with the host age. Under our conditions, irrespective of the host age, the percentage of *E. kuehniella* adults emerged was higher when one–three females parasitized simultaneously (43% of non-parasitized eggs). The percentage of developmental mortality was dependent on the number of females parasitizing simultaneously (*F*_1,36_ = 4.42, *p* = 0.04) and it was significantly higher with five females (Table 1).

The effect of *E. kuehniella* egg age (1- and 4-day-old) on the developmental time of *C. inanitus* is represented in Table 2. Regardless of the host age, the duration of the parasitoid life cycle was longer in females (*F*_1,31_ = 10.51, *p* = 0.03). The host age was also significant (*F*_1,36_ = 18.38, *p* < 0.001), and the development in mature eggs was about 3 days shorter in males and 6 days shorter in females compared to young eggs. Developmental time of *E. kuehniella* non-parasitized eggs was 43.9 ± 0.5 days from young eggs and 42.8 ± 0.5 days from mature eggs, and it was always shorter than the parasitoid cycle.

The rearing of *C. inanitus* on the factitious host had an influence on its body size, because it was significantly lower compared to the rearing in natural hosts *S. exigua* and *S. littoralis* (Table 3). Significant differences were also found between the two natural hosts, and adults had a bigger size when the parasitoid was reared in *S. littoralis* (*F*_2,18_ = 35.83, *p* < 0.001). No differences were found between sexes in the three Lepidoptera species.

### 3.2. Semi-Field Studies on the Efficacy of C. inanitus in S. exigua and S. littoralis

**Figure 1:**
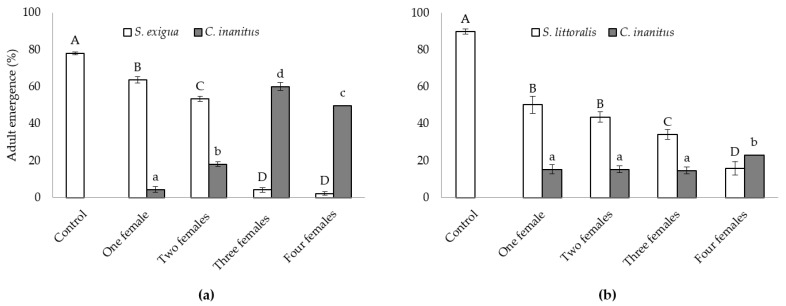
*Chelonus inanitus* parasitism success inside the greenhouse, depending on the number of females parasitizing simultaneously. Data (mean ± SEM) are percentages of noctuid and parasitoid adults emerged from 60 host eggs offered to the parasitoid. In white, host emergence; in grey, parasitoid emergence. Letters stand for significant differences among treatments (*p* < 0.05). (**a**) *Spodoptera exigua* host; (**b**) *Spodoptera littoralis* host.

Both natural hosts behaved differently concerning the parasitism success of *C. inanitus* in the greenhouse (Figure 1). The number of females parasitizing simultaneously had a significant influence on the emergence of both the host and the parasitoid in the *S. exigua* assay (Figure 1a), and only on the host emergence in the *S. littoralis* assay (Figure 1b). In the *S. exigua* assay, host emergence reached 78.1 ± 0.8% in the control treatment. Parasitization by three and four females practically suppressed host emergence (4.2 ± 1.2 and 2.5 ± 0.9%, respectively), and no statistically significant differences were recorded between those treatments. One and two parasitoid females were less effective and host emergence reached 63.6 ± 1.7% for one female and 53.3 ± 1.5% for two females (*F*_4,25_ = 729.01, *p* < 0.001) (Figure 1a). The best result concerning parasitism was achieved with three females, where *C. inanitus* emergence reached a maximum of 60.0 ± 1.2% (*F*_3,20_ = 310.58, *p* < 0.001) (Figure 1a). When the host was *S. littoralis*, the parasitoid was less effective, and reductions in host emergence were much lower than those recorded for *S. exigua* (*F*_4,25_ = 71.21, *p* < 0.001) (Figure 1b). Host emergence decreased with the number of females parasitizing simultaneously. The lowest *S. littoralis* emergence was obtained with four *C. inanitus* (15.8 ± 3.6%). Host emergence was statistically equal between one and two females. The emergence of *C. inanitus* was only statistically significant when four females parasitized simultaneously, and the number of parasitoids emerged was maximized in this treatment (23.1 ± 1.9%) (*F*_3,20_ = 3.59, *p* = 0.032) (Figure 1b).

**Figure 2:**
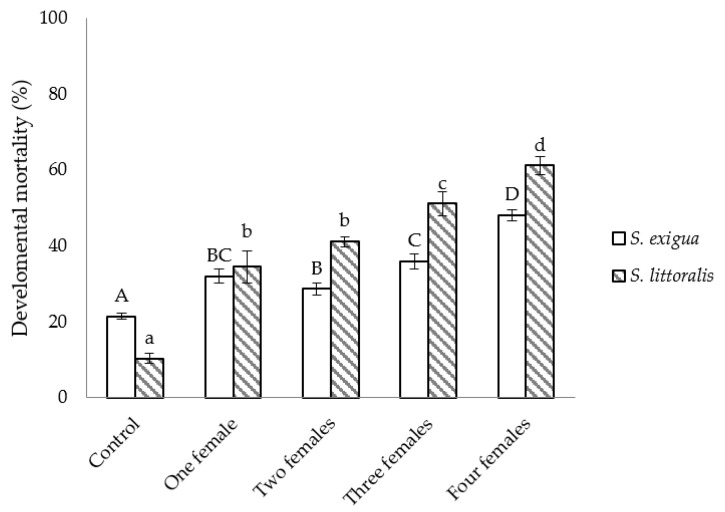
Developmental mortality of *Spodoptera* inside the greenhouse, depending on the number of *Chelonus inanitus* females parasitizing simultaneously. Data (mean ± SEM) are percentages, according to the 60 host eggs offered to the parasitoid. Letters stand for significant differences among treatments (*p* < 0.05).

The developmental mortality was higher when *S. littoralis* was the host and, irrespective of the host species, statistically higher than in non-parasitized hosts. Within each species, values tended to increase with the number of *C. inanitus* females parasitizing simultaneously (*S. exigua*: *F*_4,25_ = 39.56, *p* < 0.001; *S. littoralis*: *F*_4,25_ = 50.12, *p* < 0.001) (Figure 2).

## 4. Discussion

Many parasitoids of eggs can be successfully produced in factitious hosts at a lower cost than when they are reared in natural hosts, because less manpower and a cheaper diet are required. In koinobiont parasitoids with facultative arrhenotoky reproduction, females can potentially control the sex of the offspring by deciding if they release or not sperm as eggs pass down the oviduct (fertilized eggs developed into females and non-fertilized eggs into males). In addition, egg-larval koinobiont parasitoids such as *C. inanitus* need live host juveniles to complete their development. Therefore, there are two main problems that must be solved for the successful mass rearing of these species: (1) to decrease the excessive presence of males within a few generations in laboratory conditions [36]; and (2) to reduce the number of non-parasitized hosts because they cannot be irradiated or frozen, as is usually undertaken when idiobiontic parasitoids are mass reared (e.g., *Trichogramma* species; [25,37]). In endoparasitoid *C. inanitus*, we found that factors such as the host age offered for parasitization and the number females parasitizing simultaneously can contribute to mitigate these problems.

The egg age of the factitious host *E. kuehniella* had an influence on the *C. inanitus* female percentage in the offspring, and it was significantly increased when mature eggs (4-day-old) were parasitized. For most parasitoid species, the proportion of females in the offspring is higher in high-quality hosts [38], and may vary with host size and host age [39]. In species with facultative arrhenotoky reproduction, female eggs are bigger, but the stimuli driving the fertilization of eggs are still unknown; in addition to the species, temperature, relative humidity, photoperiod, parasitoid age and host size seem to play a role [40,41,42,43]. *Chelonus inanitus* can distinguish the host age by oviposition probing. Females lay eggs inside the embryo in mature hosts and inside the yolk in young hosts [31]. When parasitization occurs in young eggs, the parasitoid larvae must invest time and resources to reach the host embryo, which can lead to abortion. Therefore, mature eggs seem to be more suitable for parasitization because the survival of the offspring is increased [31]. However, the host age did not have a conclusive influence on the total number of *C. inanitus* emerged because the pairwise comparisons only identified a significant difference between young and mature hosts when five females parasitized simultaneously. Other authors have reported that *C. inanitus* successfully parasitizes all egg stages of the two natural hosts *S. littoralis* and *S. litura* (Fabricius) [31,44]. Generally, idiobiont parasitoids of eggs prefer young to old hosts because they obtain nutrients both from the yolk and the embryo [38]. On the contrary, koinobiont parasitoids such as *C. inanitus* barely need feeding during the first steps because they remain in the first instar until the onset of the precocious metamorphosis of the host [21,30]. The number of *Chelonus inanitus* offspring was relatively small in all treatments (below 46%), as previously reported using *E. kuehniella* as host [40], even though the parasitoid/host ratio and the parasitization times were different to those in our conditions (five or one–three females/200 eggs for 90 min vs. one female/400 eggs for 24 h). The number of *E. kuehniella* adults emerged was significantly influenced by the number of females parasitizing simultaneously. The higher the number, the more elevated the developmental mortality was (33.1 ± 3.9 and 21.4 ± 2.2, for five and one–three females, respectively), which had a positive effect on the reduction in non-parasitized hosts without reducing *C. inanitus* emergence. Superparasitism may have accounted for this [45].

*Chelonus inanitus* is a protandric species, and male developmental time is shorter than that of females. In agreement with this, the mean development duration of males was 3 days shorter than that of females in the host *E. kuehniella* (45.3 ± 0.7 and 48.3 ± 1.2 days, respectively). However, the mismatch between sexes was smaller in mature eggs (43.4 ± 0.4 days in males; 45.8 ± 1.2 days in females). In addition, because *C. inanitus* is koinobiont, it lengthened the development of parasitized hosts, as reported in some natural hosts (e.g., in *S. littoralis*; [21]). This separation between host and parasitoid emergence is positive for mass rearing because it allows the removal of host individuals prior to parasitoid emergence. In addition, shortening the parasitoid cycle by offering mature eggs for parasitization is economically beneficial for its production at a large scale.

Adult body size is one of the standardized quality parameters evaluated in mass rearing of natural enemies used in commercial biological control [46], because a smaller size has been associated with less vigor and tolerance to adverse climatic conditions [47]. For an egg-larval parasitoid such as *C. inanitus*, the adult body size may vary significantly depending on the host size during larval development. The eggs of the two noctuid species studied are similar in size (0.5–0.6 mm in diameter; [25]), but the non-parasitized larvae reach a very different size, which is much bigger in *S. littoralis* (40–45 mm; [8]) than in *S. exigua* (25–35 mm; [48]). Eggs of *E. kuehniella* are much smaller (0.35 mm; personal observation) and larvae only grow up to 12–16 mm (personal observation). In agreement with this, in our trials, *C. inanitus* body size was significantly different in the different hosts, natural or factitious, with the highest value recorded in *S. littoralis* and the lowest in *E. kuehniella*. However, the variation was under 10% among host species. 

In semi-field conditions, *C. inanitus* experienced females (one to four females; >72-h-old) reared on the factitious host *E. kuehniella*, were able to detect and parasitize eggs of two natural hosts *S. exigua* and *S. littoralis* in pepper plants. Some parasitism was even reported when only one female had to find a low number of host eggs (60) scattered in the plant. As in other *Chelonus* species, volatiles emitted by the host may have helped the parasitoid to find the eggs, in addition to physical cues such as the size and number of eggs and the host female pheromone [25,49]. The reproductive parasitism (parasitoid adults obtained/host eggs; [50]) was more successful in *S. exigua* than in *S. littoralis*. In the first host, the highest emergence of *C. inanitus* was reached when three females parasitized simultaneously, and decreased with four females, coinciding with the maximum developmental mortality. On the contrary, in *S. littoralis*, the maximum parasitoid emergence was recorded when four females parasitized simultaneously, but with a lower value than that recorded for *S. exigua*. Regarding the efficacy in the pest control, the parasitoid practically suppressed *S. exigua* adult emergence (>95%) and offered a good reduction in *S. littoralis* (around 85%). In *S. littoralis*, however, the control efficacy must be attributed to the high developmental mortality observed (non-reproductive parasitism). Generally, the efficacy of biological control is only evaluated by the parasitism rate. In most cases, the non-reproductive parasitism is underestimated even though the host probing resulting in mutilation, the pseudoparasitism (venom injection without egg laying), and the aborted parasitism can have a significant effect on the degree of parasitism success [29,31,50,51,52]. The last factor is considered to be the most important in many koinobiont parasitoids [53]. Moreover, *C. inanitus* manipulates the host development because parasitized larvae grow less than non-parasitized ones, due to the drastic reduction in food consumption since the early stages [21,30]. It has been reported that the final body weight of parasitized *S. littoralis* larvae only reaches 13% of the maximum weight of non-parasitized sixth-instar larvae [54]. Therefore, even though *C. inanitus* is an extreme koinobiont that kills the host in an advanced developmental stage, parasitized larvae cause less damage to plants.

In conclusion, based in our results, *C. inanitus* may be an interesting parasitoid of *Spodoptera* species. For the *C. inanitus* mass rearing optimization on *E. kuehniella*, mature eggs appear to be more convenient because they increased the female percentage in the offspring and shortened the parasitoid life cycle. Furthermore, a high number of females parasitizing simultaneously reduced the emergence of non-parasitized hosts. Under more realistic conditions, the parasitoid controlled both *S. exigua* and *S. littoralis*, but *S. exigua* may be a more convenient host because the parasitoid offspring was much larger. However, more realistic data on the economic suitability of a large laboratory mass-rearing using mature eggs of the hosts and a high number of females parasitizing together must be assessed to ascertain its real potential. In addition, the scarce information on *C. inanitus* performance under realistic conditions indicates that it may be highly sensitive to broad-spectrum pesticides [55]. Its release in a cotton field aiming at controlling *S. littoralis* was not very successful, and the use of the organophosphate phospholan to lower the host population was among the responsible factors [55]. Therefore, studying the compatibility between this parasitoid and the most commonly used pesticides applied in the crop is necessary before its release, a topic for which there is insufficient information in the literature.

## Figures and Tables

**Table 1:** Influence of the number of females parasitizing simultaneously (NFP) and host age (HA) on the development of *Chelonus inanitus* on the factitious host *Ephestia kuehniella* (mean ± SEM).

Parameter	NFP	HA			Statistics	NFP	HA	NFP × HA
		4-Day-Old	1-Day-Old	Mean				
*C. inanitus* adults (%)	1–3 females	37.1 ± 5.8	34.8 ± 3.9	35.9 ± 3.4	*F*	3.75	4.52	6.80
	5 females	33.9 ± 4.8 a	56.5 ± 4.4 b	45.2 ± 4.1	*p*	0.06	0.04	0.01
	Mean	35.5 ± 3.7	45.6 ± 3.8					
*C. inanitus* females (%)	1–3 females	25.2 ± 4.1	15.0 ± 4.2	20.1 ± 3.1	*F*	3.11	5.67	0.02
	5 females	31.9 ± 4.1	22.7 ± 3.9	27.3 ± 3.0	*p*	0.09	0.02	0.90
	Mean	28.6 ± 2.9 b	18.8 ± 2.9 a					
*E. kuehniella* adults (%)	1–3 females	38.9 ± 5.7	46.7 ± 5.0	42.8 ± 3.8 B	*F*	17.8	0.03	3.08
	5 females	26.8 ± 5.2	17.0 ± 3.7	21.9 ± 3.3 A	*p*	<0.001	0.84	0.09
	Mean	32.8 ± 4.0	31.8 ± 4.6					
Developmental mortality (%) ^1^	1–3 females	24.1 ± 3.2	18.6 ± 3.2	21.4 ± 2.2 A	*F*	4.42	2.15	0.16
	5 females	39.6 ± 4.9	26.6 ± 3.0	33.1 ± 3.9 B	*p*	0.04	0.15	0.69
	Mean	31.8 ± 7.1	22.6 ± 2.3					

^1^ [100 − sum of (*C. inanitus* + *E. kuehniella* adults emerged)], data were transformed to arcsin √(x/100). Developmental mortality of unexposed eggs: 10.5 ± 1.4% (young eggs); 18.4 ± 1.2% (mature eggs). Statistically significant results are in bold (*p* < 0.05). Different lower-case letters within the same row indicate differences due to host age. Different upper-case letters within the same column indicate differences due to number of females parasitizing simultaneously. All parameters have the same degrees of freedom: NFP = 1; HA = 1; error = 36; *n* = 40.

**Table 2:** Influence of the parasitoid sex and host age (HA) on the developmental time (days) of *Chelonus inanitus* grown on the factitious host *Ephestia kuehniella* (mean ± SEM).

Sex	HA			Statistics	Sex	HA	Sex × HA
	4-Day-Old	1-Day-Old	Mean				
Male	43.4 ± 0.4	46.9 ± 1.0	45.3 ± 0.7 A	*F*	10.51	18.38	1.12
Female	45.8 ± 1.2	51.5 ± 1.6	48.3 ± 1.2 B	*p*	0.03	<0.001	0.30
Mean	44.6 ± 0.7 a	48.8 ± 1.0 b					

Statistically significant results are in bold (*p* < 0.05). Different lower-case letters within the same row indicate differences due to host age. Different upper-case letters within the same column indicate differences due to sex. Degrees of freedom: Sex = 1; HA = 1; error = 31; *n* = 35.

**Table 3:** Influence of the parasitoid sex and host species (HS) on the body length (mm) of *Chelonus inanitus* adults (mean ± SEM).

Sex	HS				Statistics	Sex	HS	Sex × HS
	*S. exigua*	*S. littoralis*	*E. kuehniella*	Mean				
Male	4.61 ± 0.06	4.89 ± 0.04	4.49 ± 0.04	4.66 ± 0.06	*F*	2.72	35.83	0.38
Female	4.68 ± 0.08	5.00 ± 0.01	4.52 ± 0.07	4.73 ± 0.08	*p*	0.12	<0.001	0.69
Mean	4.64 ± 0.05 b	4.95 ± 0.05 c	4.50 ± 0.04 a					

Statistically significant results are in bold (*p* < 0.05). Different lower-case letters within the same row indicate differences due to host species. Degrees of freedom: Sex = 1; HS = 2; error = 18; *n* = 24.

## References

[1] EPPO (2015). PM 7/124 (1) *Spodoptera littoralis*, *Spodoptera litura*, *Spodoptera frugiperda*, *Spodoptera eridania*. EPPO Bull..

[2] Gabarra R., Arnó J., Riudavets J., Jacas J., Urbaneja A. (2008). Tomate. Control Biológico de Plagas Agrícolas.

[3] van der Blom J., Jacas J., Urbaneja A. (2008). Pimiento bajo abrigo. Control Biológico de Plagas Agrícolas.

[4] Robledo Camacho A., van der Blom J., Sánchez Martínez J.A., Torres Giménez S. (2009). Biological Control in Horticultural Crops.

[5] Colomer I., Aguado P., Medina P., Heredia R.M., Fereres A., Belda J.E., Viñuela E. (2011). Field trail measuring the compatibility of methoxyfenozide and flonicamid with *Orius laevigatus* Fieber (Hemiptera: Anthocoridae) and *Amblyseius swirskii* (Athias-Henriot) (Acari: Phytoseiidae) in a commercial pepper greenhouse. Pest Manag. Sci..

[6] EPPO A2 Quarantine List. https://www.eppo.int/ACTIVITIES/plant_quarantine/A2_list.

[7] CABI Invasive Species Compendium. *Spodoptera exigua* (Beet Armyworm). https://www.cabi.org/isc/datasheet/29808.

[8] CABI Invasive Species Compendium. *Spodoptera littoralis* (Cotton Leafworm). https://www.cabi.org/isc/datasheet/51070.

[9] FAUNA EUROPAEA Spodoptera littoralis. https://fauna-eu.org.

[10] OJEU (Official Journal of the European Union) (2009). Directive 2009/128/EC of the European Parliament and of the Council of 21 October 2009, establishing a framework for Community action to achieve the sustainable use of pesticides. Off. J. Eur. Union.

[11] IRAC Insecticide Resistance Action Committee. IRAC Mode of Action Classification Scheme.

[12] MAPA (Spanish Ministry of Agriculture) Phytosanitary Products Registration. https://www.mapa.gob.es/es/agricultura/temas/sanidad-vegetal/productos-fitosanitarios/registro/menu.asp.

[13] Smagghe G., Pineda S., Carton B., del Estal P., Budia F., Viñuela E. (2003). Toxicity and kinetics of methoxyfenozide in greenhouse-selected *Spodoptera exigua* (Lepidoptera: Noctuidae). Pest Manag. Sci..

[14] Cabello T., Jacas J., Urbaneja A. (2008). Control biológico de Noctuidos y otros Lepidópteros. Control Biológico de Plagas Agrícolas.

[15] Quiu B., Zhong-Shi Z., Shu-Ping L., Zai-Fu X. (2012). Effect of temperature on development, survival and fecundity of *Microplitis manila* (Hymenoptera: Braconidae). Environ. Entomol..

[16] Che W., Shi T., Wu Y., Yang Y. (2013). Insecticide resistance status of field populations of *Spodoptera exigua* (Lepidoptera: Noctuidae) from China. J. Econ. Entomol..

[17] Huang J.M., Zhao Y.X., Sun H., Ni H., Liu C., Wang X., Gao C.F. (2021). Monitoring and mechanisms of insecticide resistance in *Spodoptera exigua* (Lepidoptera: Noctuidae) with special reference to diamides. Pestic. Biochem. Phys..

[18] MAPA (Spanish Ministry of Agriculture) Phytosanitary Control Tools. https://www.mapa.gob.es/app/omdfocb/default.aspx.

[19] García-Martín M., Gámez M., Torres-Ruiz A., Cabello T. (2008). Functional response of *Chelonus oculator* (Hymenoptera: Braconidae) to temperature and its consequences to parasitism. Community Ecol..

[20] Martín Gil A., Alcázar Alba M.D., Trujillo Giménez E. (2021). Guide of Integrated Crop Protection in Solanaceae.

[21] Grossnikalus-Bürgin C., Wyler T., Pfister-Wilhelm R., Lanzrein B. (1994). Biology and morphology of the parasitoid *Chelonus inanitus* and effects on the development of its host *Spodoptera littoralis*. Invertebr. Reprod. Dev..

[22] Gauld I., Bolton B. (1996). The Hymenoptera.

[23] García-Marí F., Costa-Comelles J., Ferragut F.J. (1994). Las Plagas Agrícolas.

[24] CABI Chelonus inanitus. https://www.cabi.org/isc/datasheet/12711.

[25] Ohsaki B., Shingyouchi T., Sato Y., Kainoh Y. (2020). Host recognition by the egg larval parasitoid *Chelonus inanitus*: Effects of physical and chemical cues. Entomol. Exp. Appl..

[26] Hegazi E.M., Altahtawy M., Hammad S.M., El-Sawaf S.K. (1974). Notes on the biology of *Chelonus inanitus* (L.) (Hymen., Braconidae). Z. Angew. Entomol..

[27] Charnov E.L., Charnov E.L. (2020). Sex ratio in aged-structured populations. The Theory of Sex Allocation.

[28] Bézier A., Annaheim M., Herbinière J., Wetterwald C., Gyapay G., Bernard-Samain S., Wincker P., Roditi I., Heller M., Belghazi M. (2009). Polydnaviruses of braconid wasps derive from an ancestral Nudivirus. Science.

[29] Kaeslin M., Reinhard M., Bühler D., Roth T., Pfister-Wilhelm R., Lanzrein B. (2010). Venom of the egg-larval parasitoid *Chelonus inanitus* is a complex mixture and has multiple biological effects. J. Insect Physiol..

[30] Kaeslin M., Pfister-Wilhelm R., Lanzrein B. (2005). Influence of the parasitoid *Chelonus inanitus* and its polydnavirus on host nutritional physiology and implications for parasitoid development. J. Insect Physiol..

[31] Kaeslin M., Wehrle I., Grossniklaus-Bürgin C., Wyler T., Guggisberg U., Schittny J.C., Lanzrein B. (2005). Stage-dependent strategies of host invasion in the egg-larval parasitoid *Chelonus inanitus*. J. Insect Physiol..

[32] Lanzrein B., Hammock B. (1995). Degradation of juvenile hormone III in vitro by non-parasitized and parasitized *Spodoptera exigua* (Noctuidae) and by the endoparasitoid *Chelonus inanitus* (Braconidae). J. Insect Physiol..

[33] Pfister-Wilhem R., Lanzrein B. (2009). Stage dependent influences of polydnaviruses and the parasitoid larva on host ecdysteroids. J. Insect Physiol..

[34] Poitout S., Bues R. (1974). Rearing of lepidopteran larvae of twenty-eight Noctuidae and two Arctidae species on artificial diet. Rearing details per species. Ann. Zool. Ecol. Anim..

[35] Statgraphics Technologies Inc. (2020). Statgraphics Centurion 18.

[36] Schneider M.I., Viñuela E. (2007). Improvements in rearing method for *Hyposoter didymator* (Hymenoptera: Ichneumonidae), considering sex allocation and sex determination theories used for Hymenoptera. Biol. Control.

[37] Etzel L.K., Legner E., Bellows T.S., Fisher T.S. (1999). Culture and colonization. Handbook of Biological Control: Principles and Applications of Biological Control.

[38] Zhou Y., Abram P.K., Boivin G., Brodeur J. (2014). Increasing host age does not have the expected negative effects on the fitness parameters of an egg parasitoid. Entomol. Exp. Appl..

[39] Vinson S.B., Iwantsch G.F. (1980). Host suitability for insect parasitoids. Ann. Rev. Entomol..

[40] Rechav Y. (1978). Biological and ecological studies of the parasitoid *Chelonus inanitus* [Hym.: Braconidae] in Israel. IV. Oviposition, host preferences and sex ratio. Entomophaga.

[41] King B.H. (2002). Breeding strategies in females of the parasitoid wasp *Spalangia endius*: Effects of mating status and size. J. Insect Behav..

[42] Ode P.J., Heinz K.M. (2002). Host-size-dependent sex ratio theory and improving mass-reared parasitoid sex ratios. Biol. Control.

[43] Fuester R.W., Swan K.S., Dunning K., Taylor P.B., Ramaseshiah G. (2003). Male-biased sex ratios in *Glyptapanteles flavicoxis* (Hymenoptera: Braconidae), a parasitoid of the gypsy moth (Lepidoptera: Lymantriidae). Ann. Entomol. Soc. Am..

[44] Azam A., Kunimi Y., Inoue M.N., Nakai M. (2016). Effect of granulovirus infection of *Spodoptera litura* (Lepidoptera: Noctuidae) larvae on development of the endoparasitoid *Chelonus inanitus* (Hymenoptera: Braconidae). Appl. Entomol. Zool..

[45] Rechav Y., Orion T. (1975). The development of the immature stages of *Chelonus inanitus*. Ann. Entomol. Soc. Am..

[46] van Lenteren J.C., Hale A., Klapwijk J.N., Van Schelt J., Steinberg S., van Lenteren J.C. (2003). Guidelines for quality control of commercially produced natural enemies. Quality Control and Production of Biological Control Agents: Theory and Testing Procedures.

[47] Ameri M., Rasekh A., Michaud J.P., Allahyari H. (2013). Morphometric indicators for quality assessment in the aphid parasitoid, *Lysiphlebus fabarum* (Braconidae: Aphidiinae). Eur. J. Entomol..

[48] Azidah A.A., Sofian-Azirun M. (2007). Size of *Spodoptera exigua* (Lepidoptera: Noctuidae) larvae reared on various host plants. Malays. J. Sci..

[49] Roque-Romero L., Cisneros J., Rojas J.C., Ortiz-Carreon F.R., Malo E.A. (2020). Attraction of *Chelonus insularis* to host and host habitat volatiles during the search of *Spodoptera frugiperda* eggs. Biol. Control.

[50] Abram P.K., Brodeur J., Urbaneja A., Tena A. (2019). Nonreproductive effects of insect parasitoids on their hosts. Ann. Rev. Entomol..

[51] Kaser J.M., Nielsen A.L., Abram P.K. (2018). Biological control effects of non-reproductive host mortality caused by insect parasitoids. Ecol. Appl..

[52] Zhou J., Meng L., Li B. (2019). Non-reproductive effects of two parasitoid species on the oriental armyworm *Mythimna separata* on wheat and maize plants. BioControl.

[53] Abram P.K., Brodeur J., Burte V., Boivin G. (2016). Parasitoid-induced host egg abortion: An underappreciated component of biological control services provided by egg parasitoids. Biol. Control.

[54] Morales J., Medina P., Viñuela E. (2007). The influence of two endoparasitic wasps, *Hyposoter didymator* and *Chelonus inanitus* on the growth and food consumption of their host larva *Spodoptera littoralis*. BioControl.

[55] Rechav Y. (1976). Biological and ecological studies of the parasitoid *Chelonus inanitus* (L) (Hymenoptera: Braconidae) in Israel II. Releases of adults in a cotton field. J. Entomol. Soc. South. Afr..

